# Presence of the Neurotoxin BMAA in Aquatic Ecosystems: What Do We Really Know?

**DOI:** 10.3390/toxins6031109

**Published:** 2014-03-21

**Authors:** Elisabeth J. Faassen

**Affiliations:** Aquatic Ecology & Water Quality Management Group, Wageningen University, P.O. Box 47, Wageningen 6700 DD, The Netherlands; E-Mail: els.faassen@wur.nl; Tel.: +31-317-483-898; Fax: +31-317-419-000

**Keywords:** bioaccumulation, α-,γ-diaminobutyric acid, cyanobacteria, cyanotoxins, food web, validation, HPLC-FLD, LC-MS/MS, GC-MS, ELISA

## Abstract

The neurotoxin β-*N*-methylamino-l-alanine (BMAA) is suspected to play a role in the neurological diseases amyotrophic lateral sclerosis, Alzheimer’s disease, and Parkinson’s disease. BMAA production by cyanobacteria has been reported and contact with cyanobacteria infested waters or consumption of aquatic organisms are possible pathways to human exposure. However, there is little consensus regarding whether BMAA is present in cyanobacteria or not, and if so, at what concentrations. The aim of this review is to indicate the current state of knowledge on the presence of BMAA in aquatic ecosystems. Some studies have convincingly shown that BMAA can be present in aquatic samples at the µg/g dry weight level, which is around the detection limit of some equally credible studies in which no BMAA was detected. However, for the majority of the reviewed articles, it was unclear whether BMAA was correctly identified, either because inadequate analytical methods were used, or because poor reporting of analyses made it impossible to verify the results. Poor analysis, reporting and prolific errors have shaken the foundations of BMAA research. First steps towards estimation of human BMAA exposure are to develop and use selective, inter-laboratory validated methods and to correctly report the analytical work.

## 1. Introduction

β-*N*-Methylamino-l-alanine (BMAA) is a neurotoxin that has been linked to the progressive neurological diseases amyotrophic lateral sclerosis (ALS), Alzheimer’s disease and Parkinson’s disease [1,2,3,4]. BMAA was first discovered on the island of Guam in seeds of the cycad *Cycas micronesica* [5], which were used as food by the native Chamorro people [6]. As BMAA was shown to be neurotoxic [5], exposure to BMAA was considered as a possible cause of the high incidence of ALS/Parkinsonism-dementia complex (ALS/PDC) on this island [2]. However, the role of BMAA in the aetiology of ALS/PDC on Guam was heavily debated (e.g., [7,8,9]) and BMAA exposure is at present regarded as one of the possible causes of Western Pacific ALS-PDC [10].

BMAA research expanded beyond Guam after studies revealed the presence of BMAA beyond the seeds of the cycad, namely in the symbiotic cyanobacteria in the cycad’s coralloid roots [11,12], as well as in free living cyanobacteria unrelated to the cycad [13]. The possibility of a global presence of BMAA, and, thus, of widespread human exposure to this neurotoxin led to the hypothesis that BMAA might be related to the global presence of neurodegenerative diseases [14]. The current state of knowledge recognizes the neurotoxicity of BMAA on cellular and *in vivo* level [1,15] but an animal model for BMAA induced ALS is still lacking [15,16]. Recently, additional toxicity mechanisms have been proposed that might better explain the relation between BMAA exposure and the chronic nature of ALS/PDC [10,17]. Reports of BMAA in the brain of deceased patients suffering from ALS, PDC, or Alzheimer’s disease support the BMAA ALS/PDC hypothesis [3,18,19], however, these results could not be replicated by another research group [9,20,21]. Some, but not all, of the differences between these studies might be tracked down to the analytical procedures applied [22].

A possible important pathway for human exposure to BMAA is through cyanobacterial blooms in water or through consumption of higher aquatic organisms exposed to such blooms [13,23,24]. Recently, it was reported that also planktonic diatoms and dinoflagellates contain BMAA [25,26]. Therefore, in addition to on-going research on the role of BMAA in causing human neurodegenerative diseases, studies also focus on estimating concentrations of BMAA in aquatic ecosystems. However, reported BMAA concentrations in aquatic systems vary widely between studies. Several studies have detected BMAA in all tested cyanobacteria samples, whereas others have not detected it in any sample (Table 1). Furthermore, cyanobacterial BMAA concentrations vary orders of magnitude between studies (Table 1). Likewise, several studies have found BMAA in higher trophic levels like mollusks and fish [23,27,28,29,30,31,32,33,34], but others have not [35,36]. Bioaccumulation of BMAA in higher aquatic organisms has been reported [23]. However, BMAA concentrations in the two food web studies performed so far differ greatly: those reported for the Baltic sea (mostly ng/g dry weight (DW), [23]) were a few orders of magnitude lower than those for Florida (high µg/g up to mg/g DW [28]).

A possible explanation for the striking variations in BMAA concentrations (Table 1) could be that BMAA is produced in detectable amounts in some cyanobacteria and not in others. Concentrations of cyanobacterial secondary metabolites can vary within species, between species and between locations (e.g., [37,38,39]) but the variation within studies is usually larger than the variation between studies (e.g., [40,41])—though this is not the case for the BMAA results reported. Indeed, there is a strong bimodality in the absence/presence of BMAA in cyanobacteria samples, and analysis of similar [42,43] or comparable samples [44,45] with different methods yields different results (Table 1). This strongly suggests that additional factors to those influencing cyanobacterial metabolite production play a role in the reported divergences in cyanobacterial BMAA concentrations. In fact, the use of non-selective analytical methods likely is a major cause of the observed differences between studies [43], as is discussed in the next section. Additionally, even in cases where the appropriate analytical techniques are used, many research articles contain reporting errors such as an incomplete description of methods and results. In this setting, it is difficult to tell when BMAA has in fact been detected, as is shown in Section 3. Furthermore, the absence of critical discussions in many studies hinders the comparison of data and findings, as is shown in Section 4.

**Table 1:** Reported β-*N*-methylamino-l-alanine (BMAA) concentrations in free living cyanobacteria. Data from studies that have tested more than five independent samples are included, free and protein associated concentrations are summarized. Merged rows represent single studies. Method abbreviations are explained in Appendix information 1.

Publication year	Quantification method	Derivatization method	*n* tested samples	% positive samples	[BMAA] in positive samples µg/g DW		Ref
					average	median	
2005	LC-FLD	AQC ^§^	30	97	968	265	[13]
2008	LC-FLD	AQC	12	100	103	76	[24]
2008	LC-FLD	AQC	7	100	10	7.3	[46]
2008	GC-MS	EZ:faast	27	96	130	3.5	[44]
2008	LC-MS/MS *	none	34	0	-	-	[47]
2009	LC-MS/MS	none	21	43	13	6.0	[48]
2010	LC-MS/MS ^	none	30	0	-	-	[49]
2010	LC-MS/MS	AQC	21	100	0.01	0.01	[23]
2011	LC-MS	EZ:faast	20	80	1.4	0.49	[45]
2011	CE-UV	none	8	100	402	277	[50]
2012	LC-FLD	AQC	18	100	14	9.0	[42]
2012	LC-FLD	AQC	16	100	0.29	0.24	[42]
2012	LC-MS/MS #	AQC	8	0	-	-	[43]
2012	LC-MS/MS ~	none	8	0	-	-	[43]
2012	LC-FLD	AQC	8	38	28	22	[43]
2014	LC-MS/MS	AQC	10	100	4.4	3.2	[51]

* Limit of detection (LOD) free < 1 µg/g DW, LOD total < 4 µg/g DW; ^ LOD 1.0 µg/g DW; # LOD free 1 µg/g DW, LOD total 10 µg/g DW; ~ LOD free 0.4 µg/g DW, LOD total 1.6 µg/g DW. ^§^ AQC: 6-aminoquinolyl-*N*-hydroxysuccinimidyl carbamate.

The objective of this review is to elucidate the current state of knowledge on the presence of BMAA in aquatic ecosystems, based on studies in which appropriate analytical techniques have been employed and that were correctly reported. For this, I analyzed primary research articles on the analysis, occurrence and production of BMAA in phytoplankton and higher aquatic organisms. Moreover, in the Appendix information, I discuss some key articles on BMAA analysis, BMAA production by cyanobacteria, and human exposure through cyanobacteria to illustrate the effect of reporting errors in their context (Appendix information 2 to 6).

The main outcome of this review is that there is evidence for the presence of BMAA in aquatic organisms, but that this evidence is only based on a fraction of the published work. The assumed widespread occurrence of BMAA in aquatic ecosystems and its production by cyanobacteria could, therefore, not be verified. I find that unclear reporting and unsupported conclusions in key articles have shaken the foundations of BMAA research, an issue that needs to be tackled to determine human BMAA exposure routes and to provide a solid fundament for follow up studies.

## 2. The Role of Analytical Methods in the BMAA Controversy

The use of different analytical methods in BMAA research has recently extensively been discussed [43,52], and is summarized in this section, as it plays an important role in explaining observed differences in BMAA concentrations.

**Figure 1:**
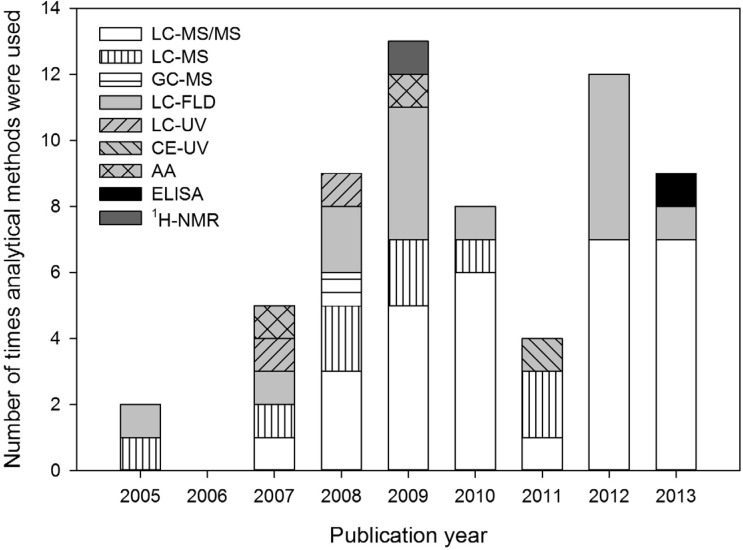
Analytical methods used for determination of BMAA concentrations in the aquatic ecosystem from 2005 up to 2013. Method abbreviations are explained in Appendix information 1.

The most selective analytical methods used for BMAA analysis are ^1^H-NMR and LC-MS/MS. ^1^H-NMR was only used in one study [53], but the sensitivity of this method is very low (LOD 5 mg/L). LC-MS/MS is the most frequently applied technique (Figure 1), and it is selective because it relies on four criteria for the identification of analytes (retention time, mass-to-charge ratio (*m/z*) of the parent ion, *m/z* of product ions after collision induced dissociation, and ratio between these product ions). Therefore, the chance of misidentification is minimized.

In LC-MS and GC-MS, no collision induced dissociation is used and these methods are, therefore, less selective than LC-MS/MS. In LC-FLD, LC- or CE-UV and AA analysis, analytes are identified by retention time and optical signal. As only two identification criteria are used by these methods, they are regarded as non-selective. An analytical technique that is based on different principles than the ones discussed before is ELISA, in which antibodies are used for identification. Recently, an ELISA for BMAA determination in environmental samples became commercially available. However, this test was shown to be unsuitable for its intended use [54].

A recent review on analytical techniques for BMAA research [52] recommends the use of MS/MS instead of optical methods (e.g., FLD) for detection. Indeed, there is a substantial chance of misidentification and overestimation of BMAA concentrations with optical detection based methods, as has experimentally been shown for one LC-FLD method [43]. In that study, FLD analyses resulted in overestimation or false positives in three out of eight tested samples. A group of scientist argued that BMAA could be successfully separated from its isomer α-,γ-diaminobutyric acid (DAB) [55] and other diamino acids [56] by a diversity of analytical methods, including those with optical detection. However, they do not properly answer the most important questions of possible misidentification by optical methods. It is unclear whether BMAA was separated from DAB in six of the ten investigated methods, because the chromatograms showing separation, or their legends are incomplete or incorrect (Appendix information 2). Furthermore, only a narrow selection of possibly interfering compounds was tested, while in real samples many more compounds could possibly interfere (Appendix information 3). Finally, it is not clear whether BMAA was correctly separated from the tested compounds in earlier studies by these authors, as it is not explicitly stated how the presented results relate to previous work (Appendix information 2 and 3).

MS/MS is currently generally accepted as the preferred detection technique for BMAA analysis, but when combined with LC separation, this technique also has its drawbacks. A main concern with LC-MS and LC-MS/MS analyses is the possible loss of signal by ion suppression [22,57], when sample components other than the analyte decrease (and in some cases also enhance) the analyte signal [58]. The severity of this effect should, therefore, be estimated and reported for each LC-MS(/MS) method [22].

To enhance its compatibility with different analytical methods, BMAA is sometimes derivatized. Derivatization is used to change the properties of BMAA, e.g., to enhance its volatility for GC-MS analysis, to add chromophores for optical detection or to reduce polarity for reversed phase LC separation. While derivatization adapts analytes to each technique, it does not necessarily influence the selectivity of each method. Therefore, the observed differences in cyanobacterial BMAA concentrations are not related to whether or not samples were derivatized (Table 1). The observed differences cannot either be explained by lack of sensitivity, as the detection limits of most methods with which no BMAA was detected were generally below the average concentrations found by others (Table 1).

Optical detection methods are still used in BMAA research (Figure 1). However, in the context of European guidelines for pesticide residue analysis, these techniques are only acceptable for frequently found residues—and always in conjunction with additional confirmatory methods—but more selective methods like MS/MS are preferred [59]. Identification by single MS is only regarded reliable when two or more diagnostic ions are used [59].

## 3. Review of Reported Methods and Results

As detailed above, BMAA can only be reliably detected if the appropriate methods are used. Furthermore, for results to be clear and comparable, it is essential to report methods and results adequately. I here evaluate the methods and results sections of studies on BMAA detection in aquatic ecosystems. For each method, I checked if the following basic information was well reported: sample origin and storage, sample processing, sample analysis, method performance and BMAA identification.

### 3.1. Sample Origin and Storage

**Figure 2:**
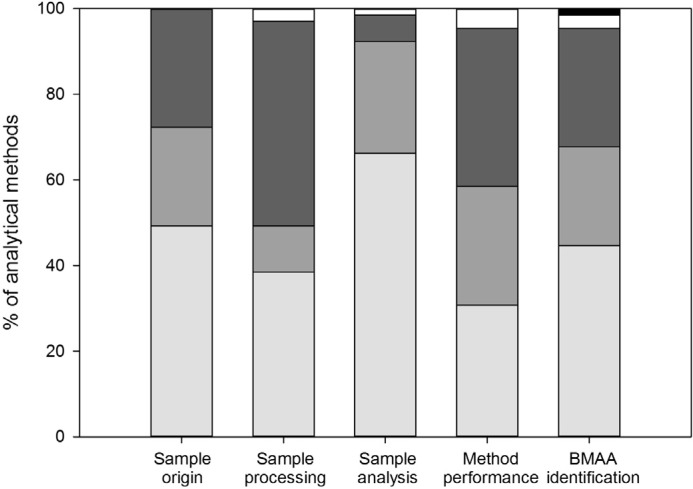
Quality of reporting for analytical methods. Bars indicate: (nearly) correct and complete (light grey), incomplete or with errors (medium grey) and absent or with major errors (dark grey). Data that have been published in previous method descriptions are shown in white, and ‘not applicable’ is shown in black. Results are summarized from Appendix information 7.

Sample origin and storage conditions were well described in nearly half of the performed analyses (Figure 2). Most of the studies for which no data on sample origin and storage were provided focused on method development, but five studies focused on BMAA detection in cyanobacteria [13,60,61,62,63]. Especially for these latter studies, information on sample origin and storage is required for the right interpretation of the detected BMAA concentrations, as cyanobacterial amino acid and toxin concentrations can change with changing growth conditions (e.g., [37,38,64,65]).

Incomplete description of sample origin in combination with incomplete data presentation can undermine the conclusions of a study. For instance, in a study on exposure of Gulf War veterans to BMAA [60], samples were taken from different locations in the Qatar desert. It is unclear how many samples were taken at each location, and which of these samples contained BMAA. The amount of BMAA in each sample was also not reported. As it unclear which locations of the Qatar desert contain which amount of BMAA, it is difficult to estimate the possible exposure of the veterans. The suggestion that BMAA exposure through desert dust may be linked to the increased incidence of ALS in Gulf War veterans is, therefore, not supported by the presented data (Appendix information 4).

### 3.2. Sample Processing

Essential information on sample processing was lacking for half of the analyses (Figure 2), and it was impossible to estimate the workup efficiency for most of these analyses. Most details were lacking on volumes and weights during sample processing and on the derivatization protocol (Appendix information 7). Information on volumes and weights is required because volume-to-weight ratios partly determine extraction efficiencies, and the amount of sample injected in LC-MS(/MS) can influence the signal strength during analysis. Furthermore, derivatization efficiency is also dependent on the sample/reagent ratio [52,66]. The derivatization procedure was only sufficiently described sixteen times, while derivatization was used in 49 analyses (Appendix information 7). For some analyses, an estimation of the total sample processing efficiency (including the derivatization step and/or cleanup) could be derived from the use of internal standards and/or recovery data (e.g., [29,44,45,67,68]). For most analyses, however, it remains unclear whether derivatization was efficient and, therefore, whether BMAA concentrations were correctly determined or underestimated.

### 3.3. Sample Analysis

In most studies the sample analysis was well described (Figure 2), but information on quantification was often missing (Appendix information 7). For 18 out of the 43 analyses in which BMAA concentrations were determined, it was unclear how this was done. Quantification can be performed in different ways, e.g., against a calibration curve of pure standards or spiked matrices, and with or without correction for internal standards or recoveries. Different methods of quantification can give different results, so this information is essential to allow comparison of studies.

When analytical methods are poorly described, they cannot be reproduced by other scientists and the results of the study cannot be validated by independent replication. For instance, some methods could not be reproduced because it was unclear how the elution programs was performed [25,69] and for another study, it was even impossible to tell which analytical procedure had been followed [33].

### 3.4. Method Performance

Method performance and validation data are used to show that the applied method is suitable for its intended purpose [70]. However, for most analyses these data were incomplete or missing (Figure 2). For instance, recovery was only correctly reported for one third of the methods (Appendix information 7). Unless internal standards are used, recovery data should be used to correct the analyzed BMAA concentration for possible losses during processing and or analysis. One of the studies for which no recovery data are available is the study on BMAA concentrations in the Baltic food web [23]. The cyanobacterial BMAA concentrations found in this study are by far lower than those found in other studies (Table 1). The validity of these results cannot be evaluated, because recovery data are neither given in the article in which the study is described [23], nor in the methodological article that preceded this study [27].

For sixteen methods, most data needed for method validation (detection limits, linear range, precision and recovery) were provided [14,27,28,32,34,36,43,45,47,50,53,68,71,72]. However, unvalidated methods (or methods for which no sufficient validation data were provided) were repeatedly referred to as ‘validated’. This was mainly the case for one LC-FLD method [14,28,30,46,55,56,62]. In addition, it was also stated that ‘the’ 6-aminoquinolyl-*N*-hydroxysuccinimidyl carbamate (AQC) based method for BMAA analysis has been validated by other methods [57]. However, it is unclear what ‘the’ AQC based method is, as AQC derivatization has been used in combination with many different analytical methods (e.g., Table 1). In the only studies where multiple AQC methods were quantitatively compared, there was a discrepancy in results between the AQC LC-FLD and the AQC LC-MS/MS method [43] and between two AQC LC-FLD methods [42].

### 3.5. BMAA Identification

Correct identification of BMAA can be shown by comparing a sample chromatogram (for methods using optical and mass spectrometry detection) or spectrum (mass spectrometry) to that of a BMAA standard or a sample spiked with BMAA. In 27 out of 65 methods, chromatograms or spectra provided enough information to prove that BMAA was correctly identified (Appendix information 7). For 21 methods, BMAA identification could not be verified because no (six methods) or only one chromatogram/spectrum was shown (fifteen methods, Appendix information 7) and the response of a sample could not be compared to that of a standard. For other methods, chromatograms were incorrectly displayed.

**Figure 3:**
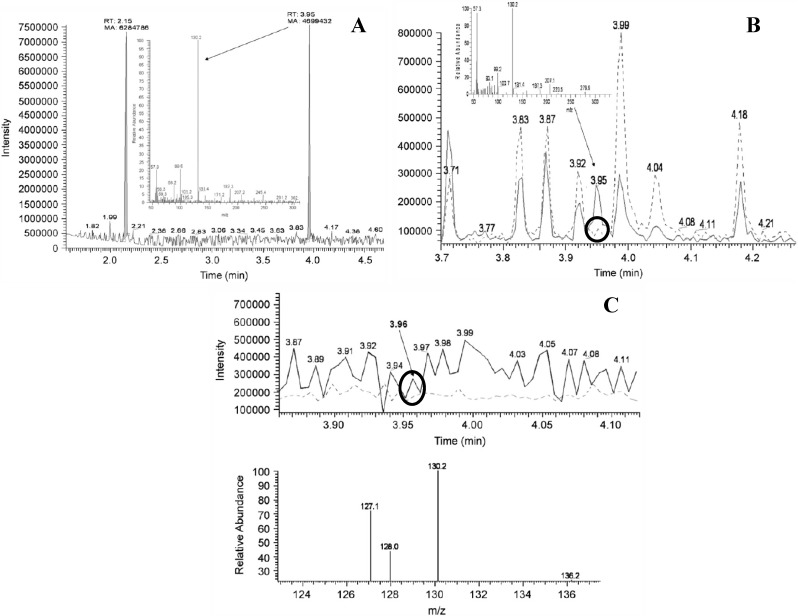
GC-MS chromatograms and spectra of standards, spiked and unspiked cyanobacterial samples as published in [44]. Circles indicate detection of unspiked BMAA and are added in this review. (**A**) Chromatogram of standard BMAA (Retention time (*R*t) 3.95 min; *m/z* 130.2) measured against an internal standard of norvaline (*R*t 2.15 min, *m/z* 158, 72); (**B**) GC-MS detection of BMAA (dotted line) detected from strain R202.1 spiked with an authenticated BMAA standard (Sigma, solid line); (**C**) Comparison of BMAA detection using a lyophilized (dotted line) culture and fresh (solid line) culture (strain Q411.1 *Leptolyngbya*). Reprinted from [44], with permission from Elsevier.

For LC-MS/MS analysis, the four analyte identification criteria (retention time, *m/z* of the parent ion, *m/z* of product ions after collision induced dissociation and ratio between these product ions) should be shown to be the same between a BMAA standard and BMAA detected in a sample. However, for many LC-MS/MS analyses this was not correctly demonstrated: none of the studies that used LC-MS/MS to confirm positive findings by LC-FLD [14,24,28,30,46,60,62,63,73] reported the LC-MS/MS identifications correctly. LC-MS/MS identification was only correctly presented in studies that used LC-MS/MS as their primary method [25,27,31,32,34,43,47,48,49,68,71,74]. Examples of incorrectly displayed LC-MS/MS identifications are spectra of standards and samples acquired at different collision energies [28,60], which makes them incomparable. Furthermore, the ratio of the product ions between the BMAA standard and the sample differed in two studies [26,30], in other studies the integration method used for the different product ions was inconsistent [29,73] and different BMAA retention times between spiked pure water and a spiked cyanobacterial extract were shown without explanation [72].

There are also problems with the proof of correct identification of BMAA in the only GC-MS study on cyanobacterial BMAA concentrations [44]. Chromatograms of standards, spiked, and unspiked samples were provided, but the peaks in the unspiked samples that were attributed to BMAA did not exceed the noise level (Figure 3B,C). Furthermore, the spectrum of the standard differed from the spectra of the samples (Figure 3), so additional fragments could not be used to confirm presence of BMAA, as is required in single MS analysis [59].

## 4. Bias through Selective Literature References and Lack of Discussion

As described in the previous sections, it is for many studies unclear whether BMAA is correctly detected and quantified. In addition, literature interpretation is hindered by a lack of critical reflection on the quality and limitations of some studies. Also, certain studies selectively cited only literature on positive findings of BMAA, thereby creating a biased view on the subject.

### 4.1. Selective Use of References

The selective use of references has in some studies resulted in a biased view towards positive findings of BMAA. For the first articles on BMAA in cyanobacteria [13,24,44], knowledge on presence of BMAA in cyanobacteria was limited and the results of these studies were in agreement with each other. However, results started to differ when no BMAA was detected in subsequent studies [47,53,75]. As a consequence, a public discussion on the suitability of the applied analytical methods and the correctness of results started (e.g., [47,49]). These conflicting data and/or methodological issues were mentioned in most articles from 2009 and later, but were ignored by some (e.g., [28,30,35,56,61,63,73]). Especially in studies in which optical detection was used as the primary analytical method and in which high BMAA concentrations were found using these methods [30,42,50,69], no reference to the debate on concentrations and methods was made.

Also in an experimental study on method performance [57], a biased view was created by selective referring to previous research. In this study, adduct formation in underivatized LC-MS analysis was investigated and it was concluded that for this type of analysis, adduct formation and complex formation may lead to an extreme underestimation of BMAA concentrations. However, the authors do not adequately discuss the underivatized LC-MS or LC-MS/MS publications in which complex and adduct formation do not seem to play a major role. Neither do they refer to the only study in which underivatized and derivatized LC-MS/MS analysis were directly compared, and in which underivatized LC-MS/MS performance was slightly better than derivatized LC-MS/MS performance. Finally, the authors recommend to use derivatized LC-MS analysis with LC-FLD as a confirmatory technique, but do not discuss the points raised against the use of LC-FLD for BMAA analysis in several other publications (Appendix information 5).

### 4.2. Discussion of Quality and Limitations of the Study

In most articles, presented work was not critically discussed and limitations of the study were rarely addressed, which contributes to uncertainty about the validity of some results. For instance, one group published an article on derivatization optimization [66], which was followed by a methodological article on SPE and LC-MS/MS analysis of samples [27]. This method was subsequently used for a food web study [23]. In 2012, the same group published two more methodological articles, one on separation of BMAA from isomers [31] and one describing quantification by LC-MS/MS [68]. These articles have greatly contributed to aquatic BMAA research, but on the same time give rise to some questions. For instance, why was an optimum ratio between sample protein and derivatization reagent advised in the first study [66], and this ratio by far exceeded in the second study [27]? Furthermore, the LC-MS/MS method was adjusted in 2012 because the ratio between product ions used for BMAA identification in samples did not always correspond to the ratio in a BMAA standard [31]. If these ratios do not correspond it is uncertain whether BMAA is present in the samples. It was concluded that the difference in ratios might have been caused by an interfering isomer, but the question regarding whether this interference was also present during the food web study [23] and, therefore, whether BMAA was correctly identified in this study was not addressed. Finally, a subsequent article by this group described an optimized LC-MS/MS method that could be used for quantification [68]. Sensitivity was improved in comparison to the first published method when expressed as fmole/injection (70 in [27] and 4.2 in [68]), but it is not discussed why, when expressed in µg/g dry weight, this method was a hundred times less sensitive (LOD of 0.1 µg/g DW) than the first method [27] by which a concentration of 0.001 µg/g DW had been detected [23].

Similarly, another group published several articles on method development and sample testing [44,45,67] and one on BMAA production by cyanobacteria [76]. In two of these articles, BMAA concentrations were determined in multiple cyanobacterial isolates. Although the tested isolates were not identical, they were described in both articles as being representative for the region and they were cultured under similar conditions [44,45]. The average BMAA concentration determined by GC-MS in one study [44] was nearly a hundred times higher than the average concentration determined by LC-MS in the other study [45] (Table 1), but possible causes of this difference were not adequately discussed [45]. Furthermore, from the experimental study [76], the authors conclude that cyanobacteria produce BMAA in response to nitrogen starvation. The authors suggest that some other studies did not detect BMAA because only nutritionally replete cyanobacteria were analyzed. However, the authors do not check this with their previous work, in which they frequently reported BMAA in cyanobacteria that were grown on BG11 [44,45], a medium that is very rich in nitrogen [77,78].

## 5. Conclusions

### 5.1. Presence of BMAA in Aquatic Ecosystems

There is evidence that BMAA can be present in cyanobacteria dominated samples [23,27,48,68,74], while in some equally credible studies, BMAA has not been detected in cyanobacteria [31,43,47,49,71,75]. The evidence for presence of BMAA is generated by studies that have used LC-MS/MS, which is at present regarded as one of the most suitable techniques for BMAA analysis due to its high selectivity and sensitivity [43,52]. In addition, these studies have correctly shown the appropriate chromatograms, so it is likely that BMAA has been correctly identified. The negative results are based on well reported LC-MS and LC-MS/MS studies. LC-MS is less selective than LC-MS/MS, but this method is included because less selective methods do not have a higher risk of creating false negative results than more selective methods.

Based on studies in which the quantification method has also properly been reported, BMAA concentrations in positive cyanobacterial samples are 0.73 µg/g DW in a cyanobacteria culture [68] or range from 4 to 42 µg/g DW in field material dominated by, but not necessarily solely comprised of, cyanobacteria [48]. These concentrations are close to the detection limits of the previously mentioned LC-MS(/MS) studies in which no BMAA was detected, which range from 0.1 to 10 µg/g DW [31,43,47,49,75] (LOD in [71] is not included because it is expressed per unit of wet weight). No BMAA was detected in cyanobacterial samples by ^1^H-NMR, another highly selective, but rather insensitive method (LOD of 5 mg/L) [53].

One LC-MS/MS based study has convincingly shown that axenic diatom cultures can contain BMAA at concentrations between 1.1 and 3.3 ng/g DW, the BMAA concentration in a cyanobacteria/diatom dominated field sample was 27.6 ng/g DW [25].

BMAA has also been detected in (some samples of) higher aquatic organisms [23,27,31,32,34], at concentrations between 4.7 and 14.1 µg/g DW in crabs [32], 6.8 and 46.9 µg/g DW in oysters [32], and 0.63 and 1.6 µg/g wet weight in mussels [34] by LC-MS/MS based studies. The two studies in which no BMAA was detected in higher aquatic organisms (fish and shrimp) have used LC-FLD analysis (LOD 0.21 µg/g DW for fish and 0.3 µg/g DW for shrimp [35,36]).

**Figure 4:**
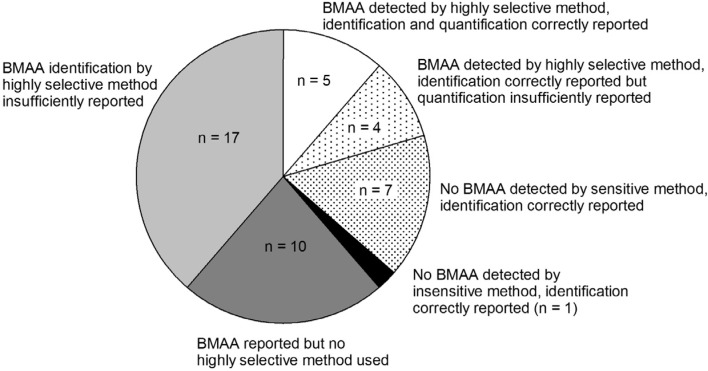
Number of studies that have provided convincing evidence for correct BMAA analysis in aquatic samples (white and dotted slices) and studies that have not provided conclusive evidence (grey and black slices). Classification is explained in Appendix information 8.

Although it is shown that BMAA can be present in aquatic ecosystems, this conclusion is only based on a narrow selection of articles (Figure 4). There is too little evidence to conclude that BMAA is occurring worldwide in aquatic ecosystems. Independent confirmation from a number of different laboratories is needed to verify this hypothesis.

More work is also needed to identify the BMAA producers in aquatic systems. Only one study has confirmed presence of BMAA in an axenic cyanobacterial culture [27]. The only study so far on BMAA production by cyanobacteria [76] does not provide evidence as the experimental setup is flawed, there is too little evidence for correct BMAA identification and not all results are presented (Appendix information 6). Diatoms might also be BMAA producers, as one study has shown that axenic diatom cultures contain BMAA [25]. Taken together, only two studies have identified cyanobacteria and diatoms as possible BMAA producers, but their findings have not yet been confirmed by other, independent laboratories. The question whether other organisms than phytoplankton are capable of BMAA production has so far only been explored for cycads [79].

For the majority of the published work on BMAA in aquatic ecosystems, it is unclear whether the results were correct. The field of BMAA analysis is still developing, and lack of certified reference material and an inter-laboratory validated method has resulted in the use of different analytical techniques. Amongst these, non-selective analytical methods have frequently been used, which may have caused false positives or overestimations. In addition, the appropriateness of some selective analytical methods could not be verified because BMAA identification was insufficiently reported.

Nevertheless, the use and description of analytical techniques are not the only sources of confusion in aquatic BMAA research. It lacks a solid foundation as the conclusions drawn in some of the key articles on analysis [55,56,57], production by cyanobacteria [76] and human exposure through cyanobacteria [60] were either unclear or not supported by the presented data. Finally, literature interpretation is hindered by occasional selective referencing to positive findings and by lack of a critical evaluation of the presented work.

### 5.2. Improving the Science

The first steps to advance BMAA research in aquatic ecosystems have been made by the increased use and development of selective analytical methods. An inter-laboratory comparison and validation of these methods, preferably using certified reference material, would be a next step toward a more unified discussion on which analytical methods to use in BMAA research. However, the field can only move forward if the use of appropriate methods is combined with correct and complete description of research. Future studies should include an extensive and correct description of methods and results (see e.g., [22]), with special attention to recovery data, quantification procedure and identification. Furthermore, the work should be critically evaluated and should be put it in its scientific context.

A solid base of knowledge relies on good insight into past results. Most evidence for correct BMAA analysis is provided in sixteen studies [23,25,27,31,32,34,35,36,43,47,48,49,68,71,74,75]. While it is well possible that BMAA has correctly been identified (and/or quantified) in other studies, there is no publicly available evidence for it. Full analysis disclosure, or availability of sample material for comparative analyses are essential to improve the current state of knowledge in BMAA research.

In conclusion, the current knowledge on presence of BMAA in aquatic ecosystems is more limited than the literature suggests. The state of knowledge will increase if appropriate, inter-laboratory validated methods are developed and used, and if the analytical work is correctly reported. This progress is needed to establish to what extent humans are exposed to BMAA through for instance ingestion of surface water during recreation and consumption of seafood. 

## Appendix

- **Appendix information 1:** Method abbreviations
- **Appendix information 2:** Article discussion ‘Distinguishing the cyanobacterial neurotoxin β-*N*-methylamino-l-alanine (BMAA) from its structural isomer 2,4-diaminobutyric acid (2,4-DAB) [55]’
- **Appendix information 3:** Article discussion ‘Distinguishing the cyanobacterial neurotoxin β-*N*-methylamino-l-alanine (BMAA) from other diamino acids [56]’
- **Appendix information 4:** Article discussion ‘Cyanobacteria and BMAA exposure from desert dust: A possible link to sporadic ALS among Gulf War veterans [60]’
- **Appendix information 5:** Article discussion ‘Reactivity of β-methylamino-l-alanine in complex sample matrixes complicating detection and quantification by mass spectrometry [57]’
- **Appendix information 6:** Article discussion ‘Nitrogen starvation results in the production of β-*N*-methylamino-l-alanine [76]’
- **Appendix information 7:** Reporting quality of methods and results
- **Appendix information 8:** Criteria used for classification in Figure 4

### Appendix information 1: Method abbreviations

**AA (amino acid analyser):**

liquid chromatography separation, detection by visible light absorption

**CE-UV:**

capillary electrophoresis separation, ultra violet detection

**ELISA (enzyme linked immunosorbent assay):**

identification by antibodies, detection by visible light absorption

**GC-MS:**

gas chromatography separation, mass spectrometry detection

**^1^** **H-NMR:**

proton nuclear magnetic resonance spectroscopy

**LC-FLD:**

Liquid chromatography separation, fluorescence detection

**LC-MS:**

Liquid chromatography separation, mass spectrometry detection

**LC-MS/MS:**

Liquid chromatography separation, tandem mass spectrometry detection

**LC-UV:**

Liquid chromatography separation, ultra violet detection

### Appendix information 2: Article discussion ‘Distinguishing the cyanobacterial neurotoxin β-*N*-methylamino-l-alanine (BMAA) from its structural isomer 2,4-diaminobutyric acid (2,4-DAB) [55]’

From 2008 up to 2010, it was repeatedly suggested that DAB had by some methods possibly been misidentified as BMAA (e.g., [47,49]). In this 2010 article, LC and GC based methods are reviewed and their ability to separate BMAA from DAB is discussed.

#### LC Separation of BMAA and DAB

The LC section starts with an overview of methods that can distinguish DAB from BMAA, and chromatograms are shown to prove separation. However, the legends of six of the ten provided chromatograms are incomplete or incorrect, so it is unclear by which methods the chromatograms are produced:

- Figure 2A in [55]: Legend refers to a 60 min gradient in [80], but in this reference a 49 minute gradient was employed.
- Figure 2C in [55]: Legend refers to studies in which no BMAA analysis was performed [81,82].
- Figure 4 in [55]: Sample used from [60], method unknown. Retention time of BMAA does not match the retention time in [60]. Figure shows BMAA and DAB, while in [60] only BMAA was shown in the LC-MS/MS chromatogram.
- Figure 6 in [55]: No method given, this seems to be a later published method [45].
- Figure 7 in [55]: No method given.
- Figure 8 in [55]: DAB not shown, no methods given but figure is identical to Figure 1 in a later published study [67]. Legend states that samples have been derivatized according to [44], but [44] is not a LC-MS but a GC-MS study in which a different derivatization procedure has been used. This is reflected by the different reported *m/z* for the BMAA derivative: 130.2 in [44] and 333 in this figure.

The authors suggest that negative findings by underivatized Hydrophilic Liquid Interaction Chromatography (HILIC) MS/MS analysis are caused by inferior performance of this method. However, they did not compare method performance data like LODs and recoveries to verify whether the performance of underivatized methods was indeed worse than that of derivatized MS/MS methods. An example is given to support the claim that HILIC separation is less efficient and results in broad peaks, however the peak that is referred to (Figure 2A in [49]) is of equal width as one of the peaks shown for derivatized separation (Figure 2A in [55]). Furthermore, authors state that DAB was routinely used in sample queues for LC methods from the first article in 2003 on [11], but in many of the articles published after 2003 by the authors, DAB is not mentioned (e.g., [13,24,46]). Furthermore, they state that negative findings of another study ([75]) were confirmed by the author’s own LC-MS/MS methods, but no method details or references are provided.

#### GC Separation of BMAA and DAB

The question whether BMAA and DAB are separated in GC based methods [44,83] is not answered in this manuscript, although there is a paragraph dedicated to this subject. It is only stated (in the LC paragraph) that in one GC-MS method [44], BMAA is separated from alanine, sarcosine and trypotophan. However, the retention times of these three amino acids have not been reported in [44]. Instead, they are taken from another study in which different conditions are used [84]. As different chromatographic conditions can lead to differences in retention times, the results of [84] cannot be extrapolated to [44]. Furthermore, it was stated that a GC-MS method developed by another group [83] might be too insensitive for BMAA detection, however only the LOD for the protein-bound fraction is referred to, and not the LOD for free BMAA, which was 50 times lower [83].

#### Discussion

In the discussion, the authors recommend a comparative exercise between laboratories that should include a repetition of validated methods (for issues with the term validated, see § 3.4 in this review), a balanced view on whether HILIC chromatography is adequate for BMAA analysis and a check for unambiguous determination of BMAA. They hereby again question the suitability of HILIC based methods without providing fact based arguments against it.

#### In Conclusion

This article suffers from many reporting deficiencies: not all addressed questions were answered, parts were suggestive and incorrect references were given. Methods that had not yet been published were included in this review, although it was stated that previous results were considered. Furthermore, separation of DAB and BMAA by GC-MS was not adequately discussed. As it is not made explicit which studies have and which have not separated BMAA from DAB, the conclusion that these compounds are “routinely and clearly distinguished by having different retention times during chromatographic separation” is not supported by the presented data.

### Appendix information 3: Article discussion ‘Distinguishing the cyanobacterial neurotoxin β-*N*-methylamino-l-alanine (BMAA) from other diamino acids [56]’

In this 2011 article, a group of scientists reports the separation of BMAA from other diamino acids. Five analytical techniques are described and chromatograms showing separation of BMAA from other compounds are presented.

#### Justification and Research Aim

It is unclear why this research has been performed, it lacks a description of the justification and aim. In the introduction, authors only state that it is important to distinguish BMAA from similar molecules. The method section does not provide clarity either. It consists of the description of five analytical techniques, all but one are not referred to, and are therefore assumed to be developed for this study. The one method referred to (LC-FLD, § 2.7. in [56]), should also be regarded as a newly developed method. Although the references [11,14,80] suggest it has already been used before, the elution program used in two of these studies differs from the one described in § 2.7. [14,80]. In the other study [11] no elution program was reported, but a reference to yet another study [85] is made. The elution program in this reference does however also not correspond to the elution program described in § 2.7.

#### Relevance of the Work

After having reached this point in the manuscript, it looks like new methods were developed to enable good separation of BMAA from other diamino acids. When placed in the context of the main debates in BMAA research from 2009 to 2011, some questions on the relevance of the work arise:

- The interference in methods based on optical detection is not limited to diamino acids, but to compounds with amino groups [43,47]. Why are only a few diamino acids tested for these methods?
- Diamino acids with a different molecular weight from BMAA are not the most likely candidates to interfere in methods with mass-spectrometry detection [43]. Why are all but two tested diamino acids compounds with a different molecular weight?

#### Relation to Previous and Future Work

In the results section, authors state that the methods have indeed been adapted in this study and show that BMAA can be distinguished from the tested diamino acids by these methods. It then, however, becomes confusing whether the subsequent conclusions are based on methods used in this study or on previously used methods. Authors state that standard LC-MS/MS methods distinguish BMAA from other amino acids, with a reference to previously used methods [14,27]. As these previous methods differ from the ones presented in this study, this statement would imply that the conclusion is based on previous methods, but this conclusion is not supported by data or chromatograms. Furthermore, it is mentioned that the BMAA isomer *N*-2(amino)ethylglycine coeluted with BMAA in the current LC-UV/MS method, but that in previous studies that included LC-UV or LC-MS analysis [14,46,60,86], BMAA identification was correct because other methods were used for confirmation. Also in this case, three [14,46,86] of the four the methods referred to are different from the tested method so it is unclear whether *N*-2(amino)ethylglycine coelution had also occurred in these studies. In the discussion “standard methods” are mentioned without definition: “We found that standard methods of amino acid analysis clearly distinguish BMAA from the twelve amino acids tested”.

The repeated referring to “standard” and “previously used” methods implies that BMAA was already separated from other diamino acids in previous work. As it is neither discussed how the methods used in this study relate to previously used methods and nor whether, to the opinion of the authors, BMAA was correctly identified in previous studies, the implications of the study remain unclear.

#### In Conclusion

It is unclear which questions are answered by this study. Five methods have been shown to separate BMAA from a selection of diamino acids, but the relevance of this selection is questionable and not discussed. As these methods have all been adapted in this study, no conclusion about separation of BMAA from the selected compounds in previous studies can be drawn. However, the repeated reference to “standard” and previously used methods could mistakenly make a reader think that it is shown in this article that BMAA was also separated from the selected compounds in previous work.

### Appendix information 4: Article discussion ‘Cyanobacteria and BMAA exposure from desert dust: A possible link to sporadic ALS among Gulf War veterans [60]’

In this 2009 article, cyanobacteria crusts from the Qatar desert were collected and analyzed for BMAA in order to assess whether veterans had been exposed to BMAA during the Gulf War. It was concluded that BMAA was present in the cyanobacteria crusts, which led to the suggestion that BMAA exposure through desert dust may be linked to the increased incidence of ALS in Gulf War veterans.

#### Incomplete Description of Methods

Cyanobacteria crust samples were taken from one location in 2007 and from three locations in 2008. It is unknown how many samples were taken from each location. After sampling, some crust samples were dried and analysed, while others were cultured and subsequently analysed. It is unclear how many samples were cultured and what the origin of this/these samples was.

Four different methods were used for BMAA analysis. It is unclear which samples were analysed by which methods. The only thing that is clear is that samples that were positive for BMAA in AA and/or LC-FLD analysis were reanalysed by LC-MS/MS and that the cultured samples were analysed by LC-MS and AA.

#### Incomplete Description of Results

Authors state in the Results section that BMAA was detected in desert crust samples from one location and that BMAA and DAB were detected in the cultured crust. It is unknown whether BMAA was detected in the samples from the other locations. The provided chromatograms do not provide more clarity as is not made clear to which samples they belong, it is only stated whether they represent dried or cultured samples. Furthermore, some samples were analyzed by multiple methods, but the results of these analyses per sample are not presented, so it is unclear whether they are in agreement. BMAA concentrations are not reported.

#### Incorrect Data Visualisation

The provided LC-MS/MS chromatogram consists of three panels, one of which gives information on the sample and the other two give information on a BMAA standard. In two of these panels, product ions for a standard and for a sample are shown. However, the product ions of the standard were acquired at different settings than those of the sample. This results in different ratios of product ions between the standard and the sample (see also § 3.5 of this review). It is unclear whether these ratios would have been the same when analysed under the same conditions and therefore whether BMAA has correctly been identified by LC-MS/MS.

#### In Conclusion

The major problem of this article is that it is unclear how many samples were analyzed and how much BMAA was present in each sample. As the presence of BMAA in cyanobacteria was linked to BMAA exposure of veterans, it is essential to know the BMAA concentrations and distribution in the sampled areas. Another problem is the confirmation by LC-MS/MS. The crust samples that tested positive for BMAA with the less selective methods LC-FLD and AA were reanalysed by the more selective method LC-MS/MS. However, the presented LC-MS/MS chromatograms do not provide enough information to prove that BMAA was correctly identified with this method.

### Appendix information 5: Article discussion ‘Reactivity of β-methylamino-l-alanine in complex sample matrixes complicating detection and quantification by mass spectrometry [57]’

This 2012 article explores a possible explanation for the frequent lack of detection of BMAA by underivatized LC-MS(/MS) analyses. For this, the effect of different solutions on the BMAA signal in LC-MS analysis is determined. The authors hypothesise that the formation of adducts and complexes hinders electrospray ionisation MS analysis and can distort chromatography.

#### Adduct and Complex Formation and the Detection of Mass-to-Charge Ratio (m/z) 119

Adduct formation is determined by adding BMAA to different trichloroacetic acid (TCA)/salt solutions. These mixtures are scanned at different cone voltages and the intensity of the ion with a *m/z* of 119 (singly charged BMAA) and the *m/z*’s of BMAA adducts and dimers are recorded. As the response of *m/z* 119 is low in most solutions and for most settings, it is concluded that even under optimal circumstances, *m/z* 119 accounts for less than 10% of the total BMAA ions in solution. This is however contradicted by Figure 2A in [57], which shows a 70% presence of *m/z* 119 and by Figure 1 in [57], which shows a 30% presence. Furthermore, this conclusion implies that other MS settings are optimized for BMAA analysis at *m/z* 119 and that TCA is the optimal solution for BMAA detection. This is contradictory to earlier work, which is not discussed in the current article. In this earlier work, *m/z* 119 is found to be the main peak after infusion of an aqueous BMAA solution [47].

Complementary to the evaluation of adduct formation, the authors explore whether metal complexes of BMAA can form in sample matrixes by chemically synthesizing a BMAA-Zinc complex. However, chemical synthesis of a BMAA-Zinc complex does not prove that this complex will be formed in real samples. To prove this, real samples should be analysed for the presence of such a complex, but this is not done.

#### Implications for Sample Analysis

The authors conclude that signal suppression and alteration of chromatographic behaviour due to adduct and complex formation may lead to an extreme underestimation of BMAA concentrations in underivatized LC-MS sample analysis, especially when external calibration curves are used for quantification. However, nearly all underivatized MS(/MS) studies have anticipated this by determining recovery [43,48,49,75,87], by using matrix based calibration curves [71] or by using D_3_BMAA as an internal standard [43,47]. In these studies, recovery rates generally exceed 80% and matrix based calibration curves have similar slopes as neat curves, so there is no indication that strong signal suppression indeed occurs in real samples. The authors, however, suggest that the reaction time used for recovery determination is too short for complex formation, and that reported recoveries are therefore highly overestimated. This is an important suggestion, as it supports the main conclusion of the article. However, it is not grounded on arguments or data. In the method section, it is not mentioned that BMAA was allowed to react with TCA or salt solutions for a given period of time, nor are data on reaction time shown in the results section. Also in an older article that describes BMAA-metal complex formation [88], no reaction times are given.

Furthermore, the authors suggest that the solutions used in this study are representative for sample extracts, but they do not provide data on metal concentrations in cyanobacterial extracts. Trace element composition of marine phytoplankton [89], however, suggest that metal concentrations in cyanobacterial extracts will be much lower than the concentrated (9 mM salts and 10 mM TCA for each treatment) solutions used in this study. The authors conclude that BMAA reactivity may complicate analysis of many different types of samples, but have not verified this conclusion by analysing real samples.

#### Recommended Analytical Procedure

The article ends with the recommendation to use derivatization combined with reversed phase chromatography for sample analysis, and to use at least two orthogonal detection methods such as FLD and MS. Finally, it is recommended to use multiple *m/z*’s in BMAA analysis. This latter recommendation is supported by the presented work, but the other recommendations are not. Firstly, no adequate comparison between derivatized and underivatized MS analysis is carried out in this study. In the only study in which these methods have been compared, derivatized MS/MS analysis did not perform better than underivatized MS/MS analysis [43], but the authors do not refer to this study in their discussion. Furthermore, FLD detection is suggested as additional technique, without referring to recent articles that provide arguments against the use of this method [43,52].

#### In Conclusion

A possible weak point in MS analysis is pointed out in this article, which is valuable. However, the relevance of the experiments for real sample analysis is not shown and most conclusions are not supported by data. Finally, not all relevant publications are discussed, resulting in a biased view on the subject.

### Appendix information 6: Article discussion ‘Nitrogen starvation results in the production of β-*N*-methylamino-l-alanine [76]’

This 2011 article was the first to investigate conditions under which cyanobacteria produce BMAA. In this experimental study, two cyanobacterial strains are repeatedly subjected to nitrogen starvation. The presence of BMAA in the cultures is monitored throughout the experiments and authors conclude that nitrogen starvation results in the production of BMAA.

#### Flaws in Experimental Setup

One weak point of this work is that the design of this experiment is flawed. Nitrogen is supplied as labelled ammonium in the experimental treatment and as unlabelled nitrate in the control treatment. As cyanobacteria differ in their response to ammonium and nitrate as nitrogen source [90], the type of nitrogen source should be similar between treatments. Furthermore, in a nitrogen starvation experiment, the proper control treatment would be nitrogen repletion rather than nitrogen starvation.

#### Suboptimal Analysis

Another problem with this study is the detection of BMAA. Samples were analysed by LC-MS/MS, either as a Q1 scan without collision induced dissociation or in multiple reaction monitoring mode with collision induced dissociation and detection of product ions. This means that the LC-MS/MS was used as a less selective LC-MS for most of the analyses.

#### Presentation of Raw Data

The presented data are either too little or too much processed for good interpretation. Examples of too little data processing are the figures in which a BMAA decrease or increase is shown. In these figures (Figure 1 and Figure 3), results are presented as LC-MS(/MS) peak areas instead of as cellular or biomass related toxin content or concentrations, which are more commonly used (e.g., [37,91]). During the experiment, a fixed volume of sample was taken at each sampling event. It is not shown how the biomass concentration changed during the experiments, but as the experiment is performed in batch cultures, it is likely to have changed. Therefore, it is reasonable to assume that different amounts of cyanobacteria were present in the different samples. As only the intensity of the MS(/MS) signal is shown, no correction is made for these changes in biomass concentration. Figure 1 and Figure 3 in [76], therefore, only reflect the total amount of BMAA present in the sample and it cannot be derived whether the observed changes in these figures reflect changes in cellular BMAA concentrations or merely result from changes in biomass concentration during the experiment.

#### Obscured Data Presentation

The changes in labelled amino acid abundance in the experimental cultures are too much processed to allow easy interpretation. The increases in labelled amino acids after nitrogen starvation are shown as ratios of the singly labelled to the unlabelled amino acids, which are subsequently normalized against control cultures. This use of ratios of ratios obscures the results. More importantly however, results do not represent a response to nitrogen starvation as the control treatment had also been nitrogen starved. The results represent the differences in cyanobacterial response to the nitrogen sources used and from the presented data it can again not be derived whether these differences are caused by changes in cellular composition and/or in biomass.

#### Incomplete Data Presentation

Not all essential data are presented. Data on biomass indicators and nutritional status of the cyanobacteria are lacking. The table that shows the increase in labelled amino acids, lacks data for some samples, but this is not explained. In addition, Figure 1 and Figure 3 only show free unlabelled BMAA, the unlabelled protein associated fractions and both fractions of labelled BMAA are missing.

#### In Conclusion

This study suffers from flaws in the experimental design and lack of data on cyanobacterial biomass, nutritional status and presence or absence of different BMAA fractions. It was for instance not checked whether the cyanobacteria were really nitrogen starved and the right control treatments were not included. Furthermore, the employed LC-MS/MS was mostly operated as a LC-MS without motivation. Too little data (e.g., chromatograms with product ions of standards and a samples) were provided to show that BMAA was correctly identified. Taken together the above mentioned flaws and omissions and either the lack of data processing or the expression of data as ratios of ratios instead, this article’s conclusions cannot be verified by the presented data.

### Appendix information 7: Reporting quality of methods and results

Methods for BMAA analysis that have been applied to environmental samples have been reviewed. For each of these methods, Table A1 shows which method details and results were reported.

**Table A1:** Reporting quality of methods and results. Only methods with which environmental samples were tested are included. Symbols used are: + (nearly) correct and complete, ~ incomplete or with errors, - absent or with major errors, p shown in previous publication of the same method, x not applicable. Colored columns summarize previous columns, colors correspond to the symbols + (green), ~ (orange), – (red) and x (white). Studies in which previously published method were used and referred to are indicated by: § [27], ^ [36], ^# ^[43] and % [25].

ref	method	Pre column derivatization	Sample origin	Growth conditions/moment of sampling	Storage conditions	Sample origin and conditions	Volumes and weights	Derivatization protocol	Processing repeatable	Hardware described	Method described	Method of quantification	Analysis repeatable	Cal curve/linearity	LOD/LOQ* defined	LOD/LOQ standard reported	LOD/LOQ sample reported	Precision	Recovery	Method performance	Chrom/spectrum standard	Chrom/spectrum sample	Chrom/spectrum spiked sample	BMAA identification
[13]	LC-MS	Y	~	-	-	**-**	-	-	**-**	+	-	x	**-**	-	-	-	-	-	-	**-**	-	-	-	**-**
[13]	LC-FLD	Y	~	-	-	**-**	-	-	**-**	~	~	~	**~**	-	-	+	-	-	~	~	+	+	-	**+**
[24]	LC-FLD	Y	+	+	+	**+**	-	-	**-**	+	+	-	**~**	-	-	+	-	-	-	**-**	+	+	+	**+**
[24]	LC-MS/MS	Y	+	+	+	**+**	-	-	**-**	+	+	x	**~**	-	-	-	-	-	-	**-**	-	~	-	**-**
[14]	AA	N	+	-	-	**~**	+	x	**+**	+	+	-	**+**	-	-	-	-	-	-	**-**	-	+	-	**~**
[14]	LC-MS/MS	Y	+	-	-	**~**	-	-	**-**	+	~	~	**~**	-	-	-	-	-	-	**-**	-	+	-	**~**
[14]	LC-MS	Y	+	-	-	**~**	~	~	**~**	+	+	x	**+**	-	-	-	-	-	-	**-**	-	~	~	**-**
[14]	LC-UV	Y	+	-	-	**~**	+	+	**+**	~	+	~	**+**	+	-	+	-	+	-	**~**	-	~	-	**-**
[14]	LC-FLD	Y	+	-	-	**~**	+	+	**+**	+	+	+	**+**	-	-	-	-	-	-	**-**	~	~	~	**~**
[66]	LC-FLD	Y	+	+	+	**+**	~	-	**-**	+	~	+	**~**	~	+	~	~	-	-	**~**	-	-	~	**-**
[75]	LC-MS	N	~	-	-	**-**	+	x	**+**	+	+	+	**+**	+	+	+	-	-	+	**+**	+	-	+	**+**
[46]	LC-UV	Y	+	+	-	**~**	~	-	**-**	~	~	-	**~**	-	-	-	-	-	-	**-**	+	+	-	**+**
[46]	LC-MS	Y	+	+	-	**~**	~	-	**-**	+	+	x	**+**	-	-	-	-	-	-	**-**	+	+	-	**+**
[46]	LC-MS/MS	Y	+	+	-	**~**	-	-	**-**	+	+	x	**+**	-	-	-	-	-	-	**-**	-	~	-	**-**
[46]	LC-FLD	Y	+	+	-	**~**	~	-	**-**	+	+	~	**+**	-	-	+	-	-	-	~	+	+	-	**+**
[44]	GC-MS	Y	+	+	+	**+**	-	-	**-**	+	+	+	**+**	+	-	+	-	-	-	**~**	+	~	~	**~**
[47]	LC-MS/MS	N	~	-	-	**-**	~	x	**+**	+	+	+	**+**	+	+	-	+	+	-	**+**	+	+	+	**+**
[53]	^1^H-NMR	X	~	-	-	**-**	~	x	**~**	~	+	+	**+**	+	+	+	-	+	+	**+**	+	-	+	**+**
[36]	LC-FLD	Y	~	-	-	**-**	+	+	**+**	+	+	x	**+**	+	+	+	+	-	+	**+**	+	+	+	**+**
[48]	LC-MS/MS	N	+	+	+	**+**	+	x	**+**	+	+	+	**+**	-	+	+	+	-	+	+	+	+	-	**+**
[61]	LC-MS	Y	+	-	-	**-**	-	-	**-**	~	-	~	**-**	-	-	+	-	-	~	~	-	-	-	**-**
[62]	LC-MS/MS	N	~	-	-	**-**	-	x	**-**	+	~	-	**~**	-	-	-	-	-	-	**-**	~	~	~	**~**
[62]	LC-FLD	Y	~	-	-	**-**	+	+	**+**	+	+	+	**+**	-	-	+	-	-	-	**-**	-	-	-	**-**
[73]	LC-MS/MS	Y	+	+	+	**+**	+	-	**~**	+	+	x	**+**	-	-	-	-	-	-	**-**	-	~	-	**-**
[63]	LC-MS/MS	Y	-	-	-	**-**	+	-	**-**	+	+	x	**+**	-	-	-	-	-	-	**-**	-	+	-	**~**
[60]	AA	N	-	+	-	**-**	-	x	**-**	+	+	x	**+**	-	-	-	-	-	-	**-**	-	+	-	**~**
[60]	LC-MS/MS	Y	-	+	-	**-**	-	-	**-**	+	+	x	**+**	-	-	-	-	-	-	**-**	~	~	-	**-**
[60]	LC-FLD	Y	-	+	-	**-**	-	-	**-**	+	~	x	**~**	-	-	-	-	-	-	**-**	+	+	-	**+**
[60]	LC-MS	Y	-	+	-	**-**	-	-	**-**	+	~	x	**~**	-	-	+	-	-	-	~	+	+	-	**+**
[87]	LC-MS/MS	N	+	+	-	**~**	~	x	**-**	+	+	x	**+**	+	+	+	+	+	~	**+**	~	~	-	**-**
[87]	LC-MS	N	+	+	-	**~**	~	x	**-**	+	+	x	**+**	+	+	+	-	+	~	**+**	+	+	-	**+**
[87]	LC-MS/MS	Y	+	+	-	**~**	~	-	**-**	+	+	x	**+**	-	-	-	-	-	-	**-**	-	-	-	**-**
[27]	LC-MS/MS	Y	+	+	+	**+**	~	+	**+**	+	+	+	**+**	-	+	+	-	+	-	**~**	+	+	-	**+**
[23]	LC-MS/MS^§^	Y	+	+	+	**+**	~	+	**+**	+	+	+	**+**	-	p	p	-	p	-	**p**	+	+	-	**+**
[28]	LC-FLD	Y	+	+	~	**+**	+	-	**~**	~	+	+	**+**	+	-	-	+	-	+	**~**	-	+	+	**+**
[28]	LC-MS/MS	Y	+	+	~	**+**	-	-	**-**	+	~	x	**~**	-	-	-	-	-	-	**-**	~	~	-	**-**
[49]	LC-MS/MS	N	+	+	+	**+**	+	x	**+**	+	+	+	**+**	-	+	-	+	-	+	**~**	+	+	-	**+**
[45]	LC-MS	Y	+	+	+	**+**	-	-	**-**	+	+	~	**+**	+	+	+	-	+	-	**+**	+	-	-	**~**
[76]	LC-MS/MS	Y	+	+	+	**+**	-	-	**-**	+	+	x	**+**	-	-	-	-	-	-	**-**	-	+	-	**~**
[42]	LC-FLD	Y	+	+	~	**+**	~	+	**+**	+	~	-	**~**	-	+	+	-	-	-	**~**	~	-	-	**-**
[50]	CE-UV	X	+	+	~	**+**	~	x	**+**	+	+	+	**+**	+	+	+	-	+	+	**+**	+	-	~	**~**
[67]	LC-MS	Y	-	-	+	**-**	~	-	**-**	+	+	~	**+**	-	-	-	-	-	+	~	-	~	-	**-**
[35]	LC-FLD^	Y	+	+	+	**+**	+	+	**+**	+	+	x	**+**	-	+	-	+	-	+	+	p	p	p	**p**
[71]	LC-MS/MS	N	+	+	+	**+**	+	x	**+**	+	+	+	**+**	+	+	+	+	+	+	**+**	+	+	+	**+**
[69]	LC-FLD	Y	+	-	-	**-**	+	+	**+**	+	-	-	**-**	+	+	+	-	~	+	**+**	+	+	-	**+**
[30]	LC-FLD	Y	+	~	+	**+**	-	-	**-**	~	+	~	**~**	-	-	+	-	-	~	~	+	+	-	**+**
[30]	LC-MS/MS	Y	+	~	+	**+**	-	-	**-**	-	+	x	**~**	-	-	-	-	-	-	**-**	~	~	-	**-**
[31]	LC-MS/MS	Y	~	-	+	**-**	~	-	**-**	+	+	x	**+**	-	+	-	+	+	-	**~**	+	+	+	**+**
[43]	LC-MS/MS	N	+	+	+	**+**	+	x	**+**	+	+	+	**+**	+	+	+	+	+	+	**+**	+	+	-	**+**
[43]	LC-MS/MS	Y	+	+	+	**+**	+	+	**+**	+	+	+	**+**	+	+	+	+	+	+	**+**	+	+	-	**+**
[43]	LC-FLD	Y	+	+	+	**+**	+	+	**+**	+	+	+	**+**	+	+	+	+	+	+	**+**	+	+	+	**+**
[32]	LC-MS/MS	Y	+	-	-	**-**	~	+	**~**	+	+	+	**+**	+	+	+	+	+	+	**+**	+	+	+	**+**
[29]	LC-MS/MS	Y	~	+	+	**~**	-	-	**-**	~	~	-	**~**	-	-	+	+	-	+	**~**	~	~	+	**~**
[68]	LC-MS/MS	Y	+	+	-	**+**	~	-	**-**	+	+	+	**+**	+	+	+	+	+	+	**+**	+	+	-	**+**
[72]	LC-MS/MS	N	+	+	+	**+**	+	x	**+**	+	+	+	**+**	+	+	+	+	+	+	**+**	+	-	~	**~**
[54]	ELISA	X	+	+	+	**+**	+	x	**+**	+	+	+	**+**	+	+	+	+	-	+	**+**	x	x	x	**x**
[54]	LC-MS/MS^#^	N	+	+	+	**+**	+	x	**+**	+	+	+	**+**	p	p	p	p	p	p	**p**	p	p	p	**p**
[33]	LC-MS/MS	Y	+	+	+	**+**	-	-	**-**	-	-	-	**-**	-	-	-	-	-	-	**-**	-	-	-	**-**
[34]	LC-MS/MS	Y	+	-	+	**~**	+	+	**+**	+	+	+	**+**	+	+	+	+	+	+	**+**	+	+	+	**+**
[74]	LC-MS/MS^§^	Y	+	+	-	**~**	p	p	**p**	+	~	x	**+**	-	-	-	-	-	-	**-**	+	+	-	**+**
[51]	LC-MS/MS	Y	+	+	~	**+**	~	~	**~**	+	~	-	**~**	-	-	-	-	-	+	**~**	-	-	-	**-**
[92]	LC-FLD	Y	+	~	+	**+**	+	+	**+**	+	+	+	**+**	+	+	-	+	-	-	**~**	-	-	+	**~**
[92]	LC-MS/MS	Y	+	~	+	**+**	+	+	**+**	+	+	-	**~**	+	+	-	+	-	-	**~**	-	-	+	**~**
[25]	LC-MS/MS	Y	+	+	+	**+**	~	-	**~**	+	~	+	**~**	+	-	-	-	-	-	**-**	+	+	-	**+**
[26]	LC-MS/MS^%^	Y	~	p	p	**+**	p	p	**p**	p	p	p	**p**	p	p	p	p	p	p	**p**	+	~	-	**~**

* Limit of detection (LOD)/Limit of quantification (LOQ).

### Appendix information 8: Criteria used for classification in Figure 4

Based on the use of selective and sensitive analytical methods, and on the reporting of BMAA identification and quantification, studies are classified into groups (Figure 4 in main text). Table A2 shows the criteria used for this classification.

**Table A2:** Criteria used for the classification in Figure 4 of the main text.

Group*	Positive results for BMAA reported	At least one highly selective method used	Sensitive method used	Identification correctly reported	Quantification correctly reported	References
1	+	+	+	+	+	[25,32,34,48,68]
2	+	+	+	+	-	[23,27,31,74]
3	-	n.a.	+	+	n.a.	[35,36,43,47,49,71,75]
4	-	+	-	+	n.a.	[53]
5	+	-	n.a.	n.a.	n.a.	[13,42,44,45,50,54,61,66,67,69]
6	n.a.	+	n.a.	-	n.a.	[14,24,26,28,29,30,33,46,51,60,62,63,72,73,76,87,92]

* Group numbers indicate: 1: BMAA detected by highly selective method, identification and quantification correctly reported; 2: BMAA detected by highly selective method, identification correctly reported, but quantification insufficiently reported; 3: No BMAA detected by sensitive method, identification correctly reported; 4: No BMAA detected by insensitive method, identification correctly reported; 5: BMAA reported but no highly selective method used; 6: BMAA identification by highly selective method insufficiently reported. n.a.: not applicable.
